# Optically anisotropic substrates *via* wrinkle-assisted convective assembly of gold nanorods on macroscopic areas

**DOI:** 10.1039/c4fd00236a

**Published:** 2015-05-07

**Authors:** Moritz Tebbe, Martin Mayer, Bernhard A. Glatz, Christoph Hanske, Patrick T. Probst, Mareen B. Müller, Matthias Karg, Munish Chanana, Tobias A. F. König, Christian Kuttner, Andreas Fery

**Affiliations:** a Physical Chemistry II , Universitätsstraße 30 , 95440 , Bayreuth , Germany . Email: andreas.fery@uni-bayreuth.de ; Fax: +49 (0)921/55-2059 ; Tel: +49 (0)921/55-2751; b Physical Chemistry I , Universitätsstraße 30 , 95440 , Bayreuth , Germany; c Institute of Building Materials , ETH Zurich , 8093 , Zurich , Switzerland

## Abstract

We demonstrate the large-scale organisation of anisotropic nanoparticles into linear assemblies displaying optical anisotropy on macroscopic areas. Monodisperse gold nanorods with a hydrophilic protein shell are arranged by dip-coating on wrinkled surfaces and subsequently transferred to indium tin oxide (ITO) substrates by capillary transfer printing. We elucidate how tuning the wrinkle amplitude enables us to precisely adjust the assembly morphology and fabricate single, double and triple nanorod lines. For the single lines, we quantify the order parameter of the assemblies as well as interparticle distances from scanning electron microscopy (SEM) images. We find an order parameter of 0.97 and a mean interparticle gap size of 7 nm. This combination of close to perfect uni-axial alignment and close-packing gives rise to pronounced macroscopic anisotropic optical properties due to strong plasmonic coupling. We characterise the optical response of the assemblies on ITO-coated glass *via* UV/vis/NIR spectroscopy and determine an optical order parameter of 0.91. The assemblies are thus plasmonic metamaterials, as their periodicity and building block sizes are well below the optical wavelength. The presented approach does not rely on lithographic patterning and provides access to functional materials, which could have applications in subwavelength waveguiding, photovoltaics, and for large-area metamaterial fabrication.

## Introduction

Bottom-up assembly of noble metal nanoparticles into well-defined structures has been a focus of scientific interest for the past decade and is expected to foster innovation in the fields of nanoelectronics,^1–3^ non-linear optics,^4–6^ photovoltaics,^7,8^ and metamaterials.^4,9–12^ In particular, assemblies from gold and silver nanoparticles have received much attention. These nanoparticles display a localised surface plasmon resonance (LSPR) for which the resonance frequency depends on the size, shape and dielectric environment of the particles. In assemblies, LSPRs of individual particles can couple, resulting in characteristic shifts of the resonance frequencies, appearance of new resonances, and modification of near fields.^13^ For these phenomena, the interparticle gap size along with the degree of order are decisive.^14^ Whereas the gap size strongly influences plasmonic coupling,^15^ the overall alignment quality is crucial for homogeneity.^14^

Amongst various approaches for ordering nanoparticles, wrinkle-assisted assembly has shown potential for scalable organisation of nanoparticles. We have first shown the linear assembly of spherical polystyrene particles into well-defined linear chains using dip-coating on wrinkle-substrates.^16^ Subsequently, the concept has been expanded to nanoparticles of varying chemical composition, size and aspect ratio.^14,16–27^ Whereas these studies proved the capability of this method for nanofabrication on macroscopic areas, the introduction of noble metal nanoparticles represents a crucial step towards surfaces with tailored functionalities.^14,17,20^ Only recently, we reported on the wrinkle-assisted assembly of protein-coated gold nanospheres into parallel chains covering centimetre-squared areas and characterised the effects of strong plasmonic coupling in these structures.^14^ Such assemblies of spherical nanoparticles are promising candidates for various applications, *e.g.* surface enhanced Raman spectroscopy,^20,28^ subwavelength waveguiding,^29,30^ or plasmon enhanced solar cells.^7,8^

In contrast to spherical nanoparticles, nanorods already possess intrinsically anisotropic optical properties.^13^ Noble metal nanorod plasmon coupling has thus drawn much attention and plenty of studies investigate interparticle coupling phenomena on a single particle level with respect to interparticle distance,^15,31^ orientation,^15,31^ aspect ratio,^31,32^ shape,^33^ template material,^34^ and nanoparticle material.^35^ The intrinsic optical anisotropy can be used to achieve more complex optical features like magnetic resonances for parallel rod structures^9–12^ or three-dimensional plasmon rulers.^36,37^ Thus, achieving regular assembly of plasmonic nanorods over large areas with high filling rates is a challenge in expanding our concept of wrinkle-assisted templating towards optical metamaterials.^14^ Many groups have put effort into developing methods for nanorod organisation. Amongst those, several have taken advantage of topographically patterned substrates in combination with convective or capillary force assembly (CFA).^1,38–42^ Recently, Rey and co-workers reported on the successful alignment of gold nanorods in poly(dimethylsiloxane) (PDMS) channels, achieving filling rates of 25% in combination with a high quality of the assemblies.^1^ However, many approaches utilise topographical patterns fabricated by top-down approaches, which are ultimately limited with respect to scalability. This bottleneck could be eliminated by wrinkle-assisted assembly.

In this work, we show for the first time that wrinkle-assisted assembly allows for the fabrication of close-packed gold nanorod assemblies covering macroscopic areas and efficient capillary transfer print onto ITO substrates. We discuss the impact of particle surface chemistry, wrinkle periodicity, and amplitude on the assembly process. Based on statistical analysis, we quantitatively investigate the degree of order and filling factor in the nanoparticle patterns, as well as the interparticle gap size distribution. Finally, we characterise the optical properties with a polarisation selective excitation. We discuss the strong polarisation dependency with respect to strong plasmonic interparticle coupling effects. These studies frame the principles for a linear assembly of plasmonic building blocks on macroscopic areas using a cost-efficient, lithography-free, and large-area self-assembly technique.

## Results and discussion

### Gold nanorod synthesis and characterisation

**Fig. 1:**
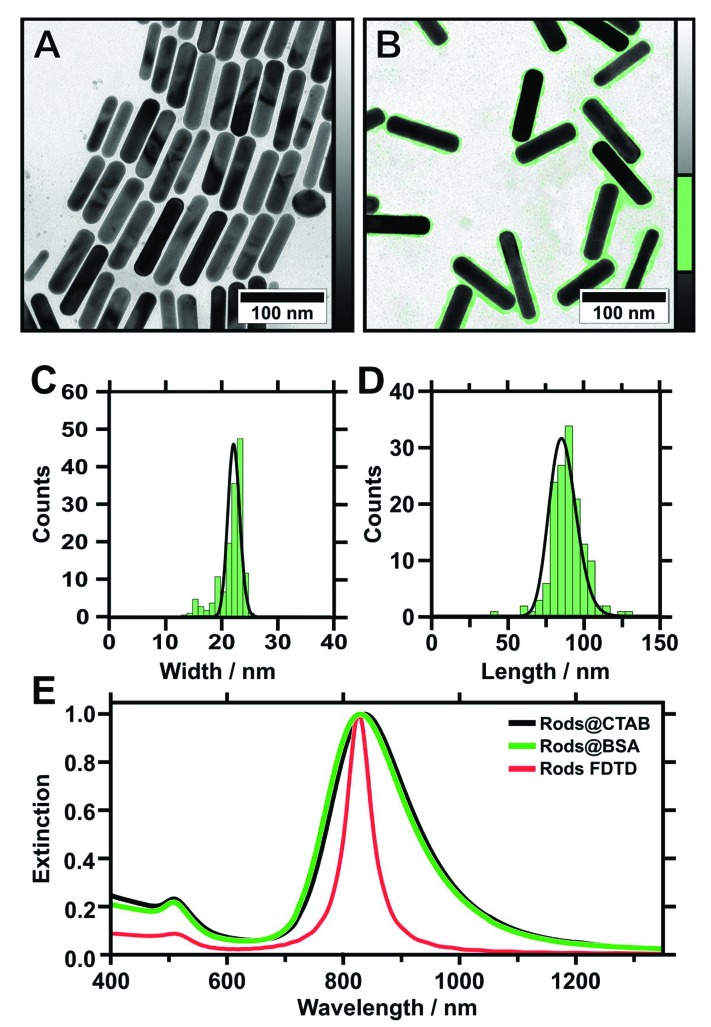
TEM images of gold nanorods stabilised with CTAB (as-prepared) (A) and stabilised with BSA after cleaning (B). The protein corona is highlighted in green colour (see adjacent colour table). Histogram plots showing the corresponding distribution of width (C) and length (D) of BSA-coated gold nanorods evaluated from TEM (investigation of 150 particles reveals: 〈width〉 = 22.0 ± 1.1 nm, 〈length〉 = 85.4 ± 9.0 nm). (E) UV/vis/NIR extinction spectra of gold nanorods in water stabilised with CTAB (black) and BSA (green) compared to the simulated extinction (red) of an 84 nm times 22 nm nanorod with 10 nm capping radius.

Monodisperse gold nanorods of high aspect ratio were synthesised using a method published by Vigderman and Zubarev in 2013.^43^ This protocol employs seeded growth of single-crystalline gold seeds in the presence of cetyltrimethylammonium bromide (CTAB) as a directing agent and hydroquinone as a rather mild reducing agent.^43^ Compared to other seeded growth protocols, which mostly employ ascorbic acid as a reducing agent, the gold conversion is significantly increased, reaching almost 100%. Furthermore, the resulting nanoparticles show a low polydispersity along with a small fraction (<1%) of particles exhibiting other shapes, such as spheres and cubes (Fig. 1A). This purity, and the remarkably narrow size distribution of the nanorods, is a prerequisite for the assemblies presented in this study. The particle size was determined by TEM (*N* = 150), yielding 85.4 ± 9.0 nm in length and 22.0 ± 1.1 nm in width (Fig. 1A–D). This results in an aspect ratio (AR) of 3.9 ± 0.6. The optical properties of the nanoparticles in dilute solution were characterised with UV/vis/NIR spectroscopy. Two characteristic plasmon modes were observed (Fig. 1E). The longitudinal mode is excited by light polarised parallel to the long axis of the particle (L-LSPR at 836 nm with a full width at half maximum (FWHM) of 220 nm), whereas the transverse mode is excited by light polarised parallel to the short axis of the nanorod (T-LSPR at 508 nm). Using a simple empiric linear equation (AR = (L-LSPR – 420 nm)/95 nm), the AR was calculated to be 4.4 ± 1.0.^43,44^ This result is in good agreement with the value determined from TEM (3.9 ± 0.6). Small deviations arise from variations of the nanorod shape (dimensions and cap rounding), which result in a blue shift of the L-LSPR and FWHM broadening.^45^ The peak intensity ratio was determined to be 4.3, serving as an indicator for synthesis quality, as spherical impurities will add up to the T-LSPR cross-section intensity. These results indicate the high quality of the synthesised gold nanorods and their narrow size distribution. Finally, we performed finite-difference time-domain (FDTD) method simulations for a single particle with the determined dimensions (TEM) to verify the quality. As shown in Fig. 4E, we observed good agreement with the experimental spectroscopic results, which ensured the full consistency of morphological and spectroscopic features on single particle level.

### Nanorod functionalisation

Previous work has shown that wrinkle-assisted assembly requires strongly hydrophilic (ideally, completely wetting) substrates.^19^ Thus, as-received nanorods are not appropriate, as they are only colloidally stable in the presence of an excess of CTAB. The amphiphilic character of CTAB however strongly influences the wetting behaviour of the nanoparticle solution on the substrate and results in a contact angle of 32 ± 0.8° for hydrophilised PDMS.

First attempts showed that the organisation of CTAB-stabilised nanorods on wrinkle-templates by dip-coating was not possible and the prepared substrates showed a poor overall coverage. Thus, further surface functionalisation is necessary to adjust the wetting behaviour of the nanoparticle solution.^14^ Consequently, CTAB was exchanged with bovine serum albumin (BSA) to adjust the nanoparticle surface chemistry and provide high colloidal stability following a recently published protocol.^14,46,47^ BSA yields a negative surface charge in basic conditions (pH > pI 4.7) as determined from zeta potential measurements and provides a defined protein corona with a thickness of 1.5 to 3 nm.^14,46,48,49^ At the same time, the nanoparticle morphology is maintained after BSA functionalisation, as we demonstrate by TEM micrographs in Fig. 1B. The functionalised nanoparticles exhibit a small shift of the L-LSPR towards blue (826 nm, FWHM 216 nm) (Fig. 1E). This shift can be attributed to negligibly small morphological alterations, due to the applied washing procedure.^47^ The resulting nanorod solutions are free of excess surfactant and can be concentrated to high gold concentrations (20 mg mL^–1^), because of the superior colloidal stability provided by the protein corona.^47,48^ For the experiments presented here, the gold concentration was 1.2 mg mL^–1^, unless stated otherwise. Thus, complete wetting of the nanoparticle solutions was achieved.

### Wrinkle-templates

**Fig. 2:**
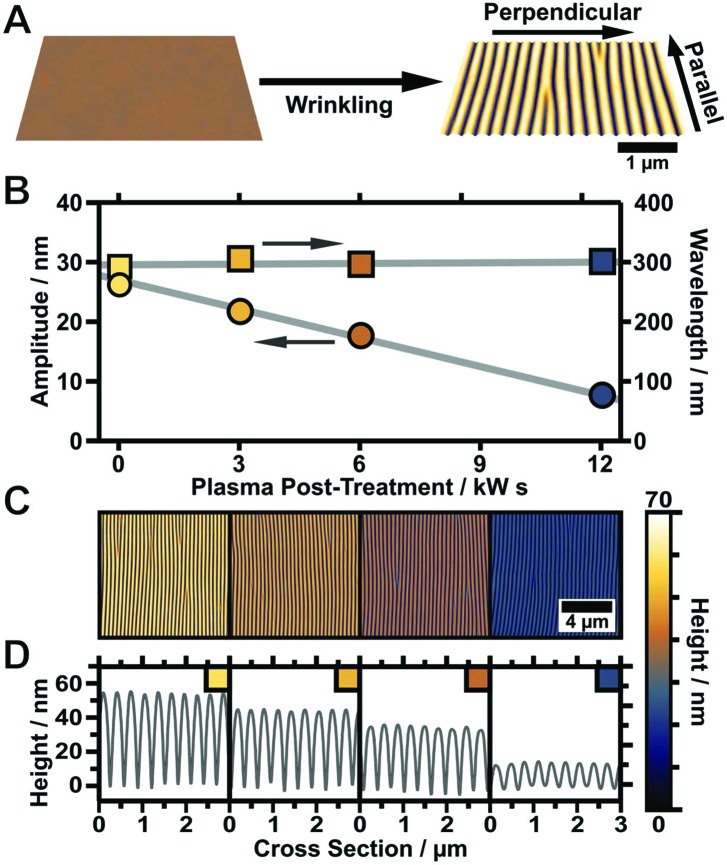
(A) Schematic depiction of wrinkle formation starting from a flat elastomeric PDMS substrate that is stretched and subsequently treated with oxygen plasma to form a thin silicon dioxide layer. Wrinkles form upon relaxation due to a mechanical mismatch between the bulk elastomer and the oxide top layer. (B) Amplitude (left axis and marked by circles) and periodicity (right axis and marked by rectangles) plotted *vs.* plasma dose of the applied post-treatment procedure (plotted lines are guides to the eye). (C) Corresponding AFM image of the wrinkled substrate after post-treatment (please note that for better comparison all images were scaled to the same height). (D) Corresponding cross-sections of the wrinkles after post-treatment, revealing a linear decrease in amplitude while the periodicity remains constant.

In order to achieve highly periodic particle arrays homogeneously distributed on large areas, the templates have to comply with two requirements. First, the nanorods require nanoscale confinement on a large area. Second, the templates should provide macroscopic and homogeneous, ideally complete wetting. The first condition is achieved with wrinkle-templates possessing periodicities close to 200 nm in combination with variable amplitudes in the range of the diameter of the nanorods. The second requirement demands avoiding cracks in the hydrophilic surface layer to prevent contact line pinning. Cracks occur parallel to the stretching directions due to the anisotropic mechanical stretching of the PDMS substrate (Fig. 2A) in combination with the mechanical mismatch of the glassy top layer and substrate.^50^ As inside the cracks hydrophobic PDMS is exposed, cracks cause local hydrophobisation as well as nonuniform wetting.

We found that the parameters listed in the experimental section in Table 1 provide crack-free substrates with wrinkle periodicities of 200 to 300 nm and wrinkle amplitudes in the range 20 to 30 nm.^51^ To render the prepared substrates hydrophilic and to gain an even higher control over surface topology, defined plasma post-treatment was employed. A variation of the plasma dose at low pressure (0.2 mbar) facilitates a precise adjustment of the wrinkle amplitude, which is tuneable from the initial amplitude down to a completely flat surface without affecting the wrinkle periodicity, as shown in Fig. 2B–D. This is a simple control parameter that enables a precise adjustment of the wrinkle aspect ratio to match the nanoparticle dimensions and directly regulates nanoparticle arrangement inside the grooves of the wrinkle-templates (template requirements). For the representative sample shown in Fig. 2 the amplitude was tuned starting from 26 nm down to 5 nm, keeping the periodicity constant at 300 nm. The presented process offers the possibility to tune the wrinkle aspect ratio (periodicity/amplitude) with a single experimental parameter, which is of great importance to match the wrinkle dimensions with building block sizes.

**Table 1:** Parameters for wrinkle preparation

Sample	Figure	Elongation (%)	Plasma time (s)	Periodicity^*a*^ (nm)	Amplitude^*a*^ (nm)
1	Fig. 3A	40	1200	276	25.2
2	Fig. 3B	60	1200	217	25.7
3	Fig. 3C & 4	40	1200	219	23.1
4	Fig. 6	40	1200	238	24.3

^*a*^Initial periodicity and amplitude.

### Nanorod assemblies

**Fig. 3:**
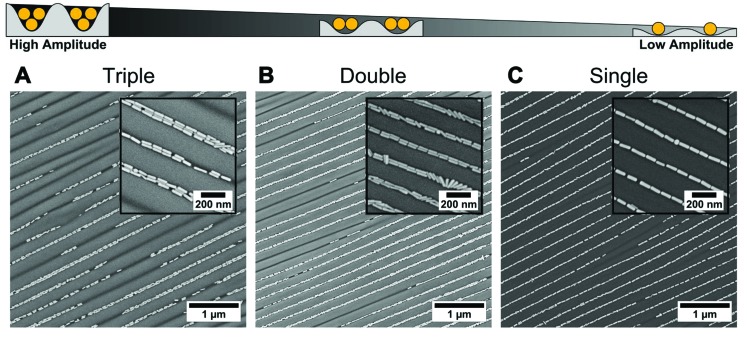
Amplitude dependency of linear assembly of BSA-functionalised gold nanorods into wrinkles using dip-coating. Organisation of gold nanorods into (A) triple lines, (B) double lines, and (C) single lines. From (A) to (C) the amplitude of the wrinkles decreases progressively. For all samples the withdrawal of the templates was performed with the wrinkles oriented perpendicular to the contact line with a withdrawing speed of 100, 10, and 10 μm min^–1^, respectively, and a gold concentration of 1 mg mL^–1^.

We use dip-coating to organise the gold nanorods into well-defined structures. As drag forces caused by the moving contact line along with convective flow foster nanorod alignment,^1^ the best results were obtained with the pulling direction oriented parallel to the wrinkles (Fig. 2A). In comparison, samples produced with a perpendicular orientation displayed a higher degree of disorder and a tendency for rod alignment parallel to the withdrawing direction (data not shown). Despite the repulsive electrostatic interaction between the (equally charged) surfaces of the particles and the wrinkles, attractive capillary forces facilitate the nanorod deposition in close-packed assemblies. Due to the confining sinusoidally shaped channel walls, remarkable site-selectivity can be achieved. The wetting behaviour of the templates is determined by the hydrophilicity of the surface, the wetting properties of the solution and the contained nanoparticles. Purified solutions of BSA-functionalised gold nanorods spread out completely on hydrophilised substrates, thus behaving similar to neat water, resulting in a contact angle close to zero. The low contact angle of the BSA solution on the receding template leads to the formation of a meniscus and thus to confinement of the gold nanorods within the receding contact line, resulting in high filling rates. For wrinkles with amplitudes slightly larger than the diameter of the anisotropic nanoparticles, the nanorods show a tendency to self-assemble into parallel triple lines, as shown in Fig. 3A. In this case, the wrinkles had a periodicity of 260 nm and an amplitude of 26.5 nm. If the wrinkle amplitude is reduced to values slightly below the nanorod diameter parallel double lines are formed preferentially, as observed for the sample with periodicity 213 nm and amplitude 15.5 nm presented in Fig. 3B. Further reduction of the amplitude, significantly below the diameter of the anisotropic building blocks, leads to a well-defined single line array of gold nanorods. This is shown in Fig. 3C for a wrinkle with a periodicity of 228 nm and amplitude of 5.2 nm. The resulting structure showed an excellent degree of selectivity, along with a good filling rate. Consequently, to promote a well-defined linear assembly of anisotropic nanoparticles in single lines on large areas, the dimensions of the wrinkle-template need to match the nanoparticle dimensions within a small range.^1^ Thus, utilising well-defined functionalised nanorods, wrinkle-templates with tuneable amplitudes and dip-coating yield a plethora of interesting particle chain morphologies, namely triple, double, and single lines. In the following, we will focus on the investigation of gold nanorods organised in single lines. This system showed the highest degree of order and homogeneity along with a good filling rate. We provide a thorough statistical analysis to quantitatively describe the degree of order and an assessment of the optical properties of single line arrays.

**Fig. 4:**
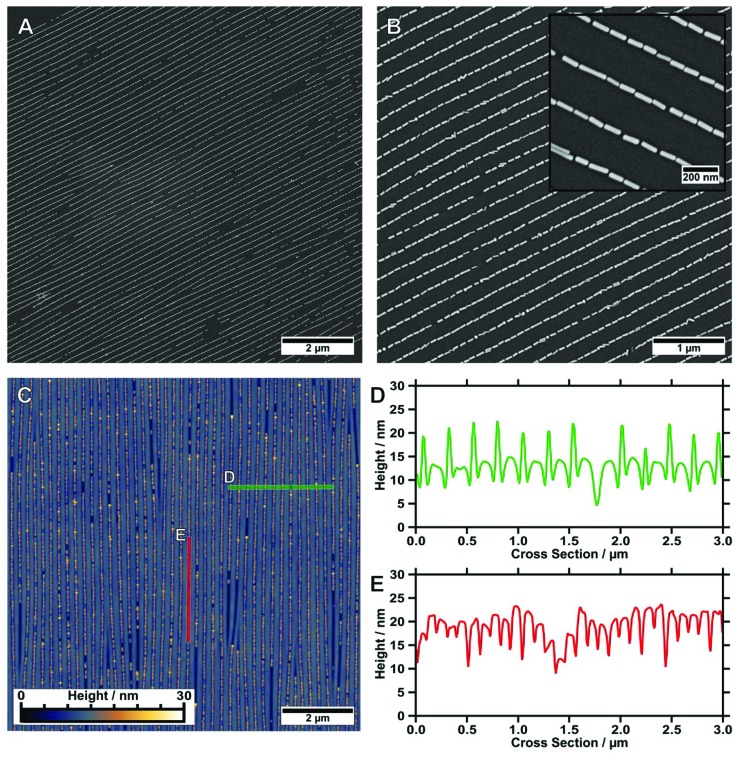
(A) Representative, low-magnification SEM image (10 × 10 μm^2^) of gold nanorods organised in single lines on a wrinkle-substrate. (B) Gold nanorods organised in single lines show a remarkably low amount of morphological impurities. The magnified inset highlights the high degree of order as well as the small interparticle gap sizes. (C) Corresponding AFM image (10 × 10 μm^2^) with cross-sections measured (D) perpendicular to the wrinkles (in green) and (E) along the channel direction (in red).

**Fig. 5:**
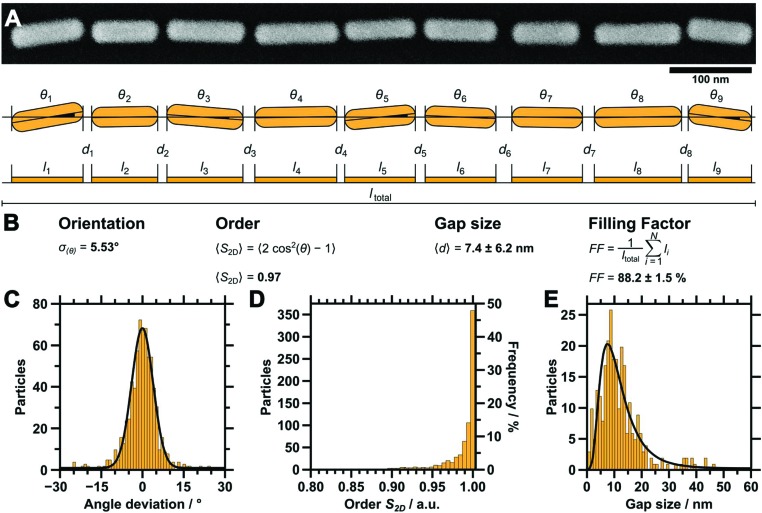
(A) High magnification SEM image of an example single nanorod line (top) and a schematic depiction of the parameter evaluation (bottom). The angle deviations *θ* of each single particle, along with the interparticle gap distances *d*, were determined with a semi-automated procedure (IGOR Pro, Wavemetrics). (B) Summary of the equations used for analysis. (C) Distribution of the deviation from the mean angle of individual particles (bin size 1°, Gaussian fit, *σ*_〈*θ*〉_ of 5.53°). (D) Distribution of the order of orientation as determined from the angle deviation (bin size 0.05, 〈*S*_2D_〉 = 0.97). (E) Gap size distribution (bin size 1.117 nm, LogNormal fit, 〈*d*〉 = 7.4 ± 6.2 nm). The filling factor was determined to be 88.2 ± 1.5%.

In order to study the homogeneity of the assembled gold nanorod arrays and structural parameters such as periodicity and filling factor, we recorded a series of SEM images with different magnifications at different points on the substrate. Representative SEM micrographs are shown in Fig. 4. The SEM micrograph with lower magnification confirms the high degree of selectivity. On the investigated 10 times 10 μm areas (Fig. 4A), no rods were identified outside the wrinkles. Furthermore, we found a high degree of filling along with well-defined orientation parallel to the wrinkles over the whole area. Fig. 4B shows an SEM image measured with higher magnification, highlighting the close packing of the nanorods organised in single lines. The AFM measurements shown in Fig. 4C and the cross-sections in Fig. 4D–E confirm a wrinkle amplitude smaller than the particle diameter. In order to quantitatively assess the quality of the arrays, we determined the overall order parameter *S*_2D_, the interparticle gap size and the one-dimensional filling factor using a semi-automated IGOR Pro procedure (see Experimental section). As shown in Fig. 5A for a representative higher magnification SEM image, SEM images were first converted to binary data, which allowed precise tracing of the boundaries of the particles. The preferred orientation and deviations from this, as well as the fill factor and interparticle distances, could thus be evaluated, as described in detail in the experimental section. Fig. 5B summarises the results for these parameters. The determined angle deviations for individual nanorods are plotted as a histogram in Fig. 5C. A Gaussian fit provides a standard deviation *σ*_〈*θ*〉_ of 5.53°. To quantify the degree of the order of orientation we determine the averaged two-dimensional order parameter 〈*S*_2D_〉 from the angle deviation *θ* of individual nanorods, according to *S*_2D_ = 2 cos(*θ*)^2^ –1. For an ideal structure, one expects an order parameter of unity, whereas a completely disordered structure would result in an order parameter of zero. Our macroscopic assemblies allow us to evaluate 〈*S*_2D_〉 for the first time with high statistical significance. *S*_2D_ was determined for more than 700 particles on a sample area larger than 15 μm.^2^ The resulting values are plotted in Fig. 5D. Consequently, the averaged two-dimensional order parameter 〈*S*_2D_〉 was determined to be 0.97. This further validates the high degree of structural control with our process. Interparticle gaps were measured to determine the mean gap size and the overall filling factor. The results for the gap sizes are plotted as a histogram in Fig. 5E. The applied LogNormal fit yields a mean gap size of only 7.4 ± 6.2 nm. The filling factor was determined as 88.2 ± 1.5% within the analysed area. The low average interparticle gap size will result in plasmonic coupling of adjacent nanoparticles as it is well below the dimensions of the individual constituents. At the same time, the high degree of filling should ensure that the effects are strong enough to be detectable in macroscopic UV/vis/NIR spectroscopy. Therefore, we proceeded with the transfer to flat substrates and spectroscopic characterisation.

### Capillary printing transfer and spectroscopic characterisation

**Fig. 6:**
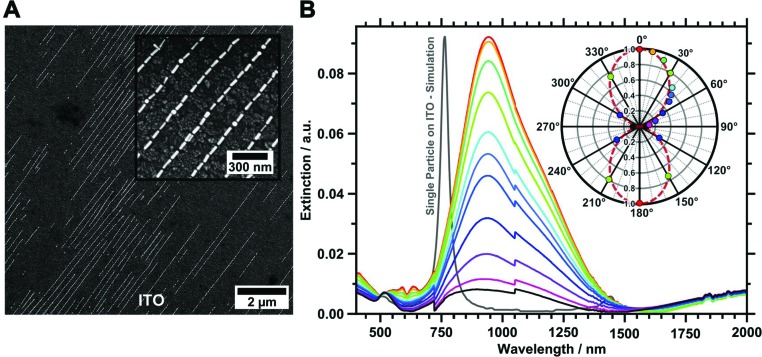
(A) SEM of gold nanoparticles transferred onto a flat ITO substrate surface functionalised with PEI. (B) Polarisation angle-dependent UV/vis/NIR extinction spectra were recorded from 0° (red) to 90° (black) in 10° steps including 45°. The normalised cross-section intensity of the L-LSPR is plotted as a polar plot (inset). The dashed red line represents a fitted cosine squared function.

To characterise the gold nanorod arrays optically, we transferred the single line assemblies onto transparent ITO-coated glass substrates. Besides its transparency, ITO provides electric conductivity, which allows for high magnification SEM characterisation without previous application of a conducting layer. Thus, the samples can be characterised with SEM prior to UV/vis/NIR measurements. Transfer onto flat ITO substrates, cleaned by the RCA SC-1 method^52^ and functionalised with a PEI layer for enhanced adhesion, was conducted as described before.^14^ A 5 μL droplet of water (pH 10) was placed between the organised films and the substrate promotes transfer from the hydrophobic wrinkle-substrate to the hydrophilic ITO surface driven by capillary forces. To ensure conformal contact between the wrinkles and the ITO target surface, the sample was pressed (140 kPa) onto the substrate using a metal block. This method is highly flexible and can be employed for transferring organised nanorods onto almost every target substrate, as long as the substrate is flat and hydrophilic, *e.g.* gold and glass samples.^14,19^ To verify the successful transfer, both the ITO substrate and the residual wrinkles were analysed by SEM and AFM. SEM images of the transferred structure show the transfer to the conductive substrate as displayed in Fig. 6A. Extinctions were measured with angles of polarisation between 0° (parallel to organised nanorod lines) and 90° (perpendicular to organised nanorod lines). The resulting extinction spectra are plotted in Fig. 6B, and show strong polarisation dependency, along with a complex plasmonic mode structure. The most prominent spectral feature is a pronounced longitudinal resonance in the range 750–1500 nm. This resonance is characterised by a dominant peak at 941 nm along with a small shoulder around 1200 nm.

The extinction cross-section intensity of the longitudinal mode is strongly polarisation dependent. Thus, the highest extinction intensity is observed for excitation polarised parallel to the linear assembled nanorods. For light polarised perpendicular to the orientation of the gold nanorods a transversal mode, slightly shifted towards the red compared to particles in solution, is observed. The longitudinal mode is strongly reduced for this polarisation, resulting in an extinction cross-section of comparable intensity, as observed for the transversal mode. The measured extinction spectra clearly reveal that the well-aligned gold nanorods render the sample optically anisotropic. This significant effect, characterised by a strong decrease of the L-LSPR peak height, is summarised in the polar plot in the inset in Fig. 6B also including data measured for angles larger than 90°. The data shown in the polar plot were normalised to the peak maxima of the longitudinal mode at an angle of polarisation of zero degrees. The changes in peak intensity are well described with a cosine square function. We used the equation *S*_Optical_ = 1 – (*A*_⊥_/*A*_‖_) to determine an optical order parameter *S*_optical_ of 0.91.^53,54^ to the best of our knowledge, such a remarkably high optical order obtained from self-assembled anisotropic colloids has not been reported before.

FDTD modelling of a single nanorod located on an ITO substrate predicts the single particle L-LSPR at 761 nm as shown in Fig. 6B. Thus the experimentally found strongly red-shifted L-LSPR is clearly not a single particle feature, but is due to strong coupling of multiple particles.^14,15,45^ However, the detailed investigation of the observed fine structure of the plasmonic coupling modes will be subject of a future study.

## Conclusion

We presented an efficient method to prepare macroscopic arrays of well-aligned gold nanorods, which can be transferred to transparent substrates. The applied multistep procedure includes the synthesis of monodisperse gold nanorods as anisotropic building blocks, followed by a surface functionalisation, which allows the elimination of the present surfactant while keeping the nanoparticles colloidally stable. Thus, the particles become compatible with wrinkle-assisted assembly on hydrophilic substrates. We demonstrated the preparation of triple, double and single line structures by dip-coating. The key parameter for organising anisotropic nanoparticles into well-defined structures was found to be the amplitude of the wrinkle-templates, which can be tuned *via* variation of the plasma post-treatment duration. This parameter offers control over the structure type, finally enabling the strong confinement of nanorods in single lines with small average interparticle distances. Evaluation of SEM images for single lines revealed an extremely high averaged two-dimensional order parameter 〈*S*_2D_〉 with a value of 0.97. This finding correlates well with the resulting anisotropic optical behaviour, yielding an optical order parameter *S*_Optical_ of 0.91. For self-assembled nanorods such high order parameters on macroscopic areas have not yet been reported in the literature. Furthermore, the nanorod double lines have great potential for the preparation of bottom-up self-assembled optical metamaterials. Such assemblies could provide an effective negative refractive index, as not only the permittivity but also the permeability should be below zero according to the literature.^12^ However, the presented multi-hierarchical structures already represent metamaterials, as the effective optical properties are located in a spectral range 5 times larger than the periodicity and 10 times larger than the individual building blocks.^55,56^ In consequence, the presented functional materials might find application in various emerging fields such as optical filters, waveguiding, non-linear optics, and metamaterials.

## Experimental

### Materials

Silver nitrate (AgNO_3_, 99.9999%), sodium borohydride (NaBH_4_, 99%), hydroquinone (HQ, >99%), hydrogen tetrachloroaurate (HAuCl_4_, >99.9%), ammonia hydroxide solution (25%), hydrogen peroxide (30%), polyethylenimine (PEI, 25 000 g mol^–1^, highly branched), ITO substrates (roughness (*R*_a_) of 2.68 nm, resistance of 8–12 Ω sq.^–1^) and bovine serum albumin (BSA, 98%) were purchased from Sigma Aldrich. Citrate (99%) and 1 M NaOH solutions were supplied by Gróssing. Cetyltrimethylammonium bromide (CTAB, 99%, 0.359 mg kg^–1^ iodine) was received from Merck KGaA. Sylgard 184 PDMS elastomer kits were purchased from Dow Corning. All chemicals and solvents were used as received. Pure-grade solvents and Milli-Q-grade water were used in all preparations.

### Gold nanorods

The protocol for the preparation of single crystalline gold nanorods was adopted from Vigderman and Zubarev.^43^ Briefly, single crystalline seeds were prepared by the addition of 5 mL of 0.2 M aqueous CTAB solution to 5 mL of an aqueous 0.5 mM HAuCL_4_·3H_2_O solution (47.3 μL of a 0.10569 M HAuCl_4_·3H_2_O stock solution). The reaction was initiated under rapid stirring at 1200 rpm by the injection of 600 μL of a 0.01 mM freshly prepared NaBH_4_ solution. Stirring was continued for 2 min followed by aging of the seeds at 32 °C for 30 min without stirring. For the growth solution, 1183 μL of an aqueous 0.10569 M solution of HAuCl_4_·3H_2_O (f.c.: 0.5 mM) was added to 250 mL of a 0.1 M aqueous CTAB solution. Followed by the addition of 500 μL of a 0.1 M AgNO_3_ concentration (f.c.: 0.2 mM). After shaking, 12.5 mL of an aqueous solution containing 0.1 M hydroquinone (final concentration is 5 mM) was added. After the mixture became clear, 3 mL of the seeds was added under rapid stirring at 1000 rpm and the solution was stored at 32 °C overnight.

### BSA functionalisation

BSA functionalisation was performed by concentrating 250 mL gold nanorods to 40 mL by centrifugation at 4000 RCF. Subsequently, 10 mL of this stock solution was used for further functionalisation. First, the CTAB concentration was set to 1 mM. Directly before functionalisation, the CTAB concentration was reduced to 0.1 mM followed by the addition of the 10 mL nanorod solution to 30 mL of a 10 mg mL^–1^ BSA solution containing 0.02 wt% citrate. The solution was sonicated for 20 min and centrifuged at 3000 RCF. After removal of the supernatant, 20 mL of a 1 mg mL^–1^ BSA solution containing 0.02 wt% citrate (adjusted to pH 10) was added. The nanoparticles were incubated overnight in a fridge at 8 °C. Functionalised nanorods were washed at least 4 times at 2500 RCF with Milli-Q water with pH 10 adjusted with 1 M NaOH.

### Wrinkle-templates

PDMS was prepared by casting 25 g of a well-dispersed (1 : 5 ratio) cross-linker/pre-polymer mixture into a balanced rectangular polystyrene dish. The plastic dish was covered to prevent the PDMS from impurities. Subsequently, the PDMS was cross-linked at RT for 24 h, followed by cross-linking at 80 °C for 5 h. Next, the cross-linked elastomer was cut into pieces of size 1 × 4.5 cm^2^, and stretched to the desired value in a home made stretching apparatus. By plasma treatment, with an oxygen pressure in the range 0.6–1.2 mbar, a thin stiff SiO_2_ layer was applied.^51^ Upon relaxation, wrinkles were formed on the surface of the PDMS. The applied plasma-treatment parameters are summarised in Table 1.

### Post-treatment

Wrinkle-templates were cut into pieces of size 7 × 10 mm^2^. To remove dust, the templates were sonicated in Milli-Q water at RT for 15 s and dried under a N_2_ stream, followed by applying a low-pressure (0.2 mbar) oxygen plasma with controlled post-treatment parameters, as summarised in Table 2. Glass slides of size 8 × 100 mm^2^ were cut from commercially available glass slides and were sonicated with Milli-Q water at RT for 15 s. Next they were washed with EtOH and dried under a N_2_ stream. The glass slides were plasma activated together with the wrinkle-templates. Afterwards, the wrinkle-templates were placed onto the glass to fix them covalently. The wrinkles were always orientated parallel to the major axis of the glass slides.

**Table 2:** Parameters for post-treatment process

Sample	Figure	Post-treatment time (s)	Periodicity^*a*^ (nm)	Amplitude^*a*^ (nm)
1	Fig. 3A	30	260	26.5
2	Fig. 3B	30	213	15.3
3	Fig. 3C & 4	60	228	5.2
4	Fig. 6	30	233^*b*^	7.7^*b*^

^*a*^Final periodicity and amplitude.

^*b*^Values were determined for transferred nanorods on ITO.

### Dip-coating

Dip-coating was performed on fixed wrinkle-templates. The as-prepared samples were clamped into the dip-coater with the wrinkle-templates at the bottom end. UV/vis/NIR glass cuvettes with a size of 10 × 10 mm^2^ were used as containers for the gold solutions. A volume of 500 μL with a gold concentration of 1.2 mg mL^–1^ (if not stated differently) was used for the dip-coating experiments. The samples were quickly immersed into the solution followed by slow removing with 10 μm min^–1^ (if not stated differently). All parameters used for dip-coating are summarised in Table 3.

**Table 3:** Parameters for dip-coating

Sample	Figure	Particle concentration (mg mL^–1^)	Orientation (°)	Withdrawing speed (μm min^–1^)
1	Fig. 3A	1.2	0	100
2	Fig. 3B	1.2	0	10
3	Fig. 3C & 4	1	0	10
4	Fig. 6	0.8	0	10

### RCA cleaning

RCA SC-1 cleaning was performed following a simplified protocol based on a procedure published by Kern and Puotinen.^52^ Briefly, the ITO substrates were cut into pieces of 10 × 20 mm^2^ and ultrasonically treated for 20 min in an isopropanol–water mixture with a ratio of 3 : 1 (v/v), rinsed with water, and placed in a bath containing an NH_3_ (25%)–H_2_O_2_ (30%)–water mixture with a ratio of 1 : 1 : 5 (v/v/v) at 80 °C for 15 min. The cleaned substrates were rinsed with Milli-Q water and stored in a water bath until their usage.

### Transfer

The transfer of the organised gold nanorods in wrinkles onto ITO substrates was performed following a protocol published recently.^14^ Briefly, the cleaned ITO substrates were coated with a PEI layer by placing them in an aqueous solution containing 10 g L^–1^ PEI. After 30 min the ITO substrates were rinsed with water and dried under a N_2_ stream. The functionalised substrates were directly used for transfer. A 5 μL droplet of water (pH 10) was applied on the surface. Next, the wrinkle-templates were placed on the surface and for better adhesion pressed onto the ITO surface using a metal bar with an effective pressure of 140 kPa. After 4 h, the transfer was accomplished and the wrinkle-template was removed.

### Instrumentation and data evaluation

#### TEM measurements

TEM measurements were performed on a Zeiss 922 OMEGA EFTEM at a voltage of 120 kV. Zero-loss filtered images were recorded using a bottom mounted Ultrascan 1000 (Gatan) CCD camera system. Gatan Digital Micrograph 3.9 for GMS 1.4 software was used for image acquisition. TEM samples were concentrated and in the case of CTAB the surfactant concentration was set to 1 mM. Droplets of 2 μL were dried on Quantifoil 300 mesh copper grid with carbon films. For size evaluation the software ImageJ (version 1.44p, U.S. National Institute of Health) was used. For the determination of the mean particle diameters 150 particles/sample were measured.

#### UV/vis/NIR measurements

UV/vis/NIR spectra were measured with a Cary 5000 spectrophotometer (Agilent, USA) with an attached Cary Universal Measurement Accessory (UMA). For film measurements, first, background spectra for all angles were recorded on the sample at a location without structure. All spectra were recorded with a slit width set to 1 nm for UV/vis and 4 nm for NIR with an integration time set to 0.2 s resulting in 300 nm min^–1^ recorded over a spectral range from 350 to 2500 nm. The measured spot size on the substrate was 4 × 4 mm^2^. All angles were measured in steps of 10° including 45°; beyond 90° the step size was set to 30°. The presented extinction spectra were calculated by dividing the transmission spectra of the sample by the transmission spectra of the background, followed by applying a negative logarithm to create extinction spectra. Due to slight inconsistency of the measured background and the background at the structure, the baseline corrected transmission spectra exhibited a value above 100% for parts of the spectral range (note these artefacts did not occur within the range of the peak position). This resulted in physically not meaningful negative extinction for small spectral ranges. To correct this artefact, all extinction spectra were shifted by a fixed value (0.014), which did not affect the relative extinction values. Additionally, due to the low overall extinction, changeover artefacts are apparent. However, we decided not to correct the spectral data with respect to these artefacts and show the raw data instead. The authors are aware that, as a consequence, the calculated peak height is not fully consistent and the anisotropy might be slightly overestimated for angles above 45°.

#### AFM measurements

AFM images were obtained using two different commercial atomic force microscope instruments, a Nanoscope Dimension V and a Dimension Icon atomic force microscope (AFM) from Bruker, USA, operated in TappingMode™. Al-coated silicon cantilevers (OTESPA, Bruker), with a stiffness of typically 35–47.2 N m^–1^ and typical resonance frequencies of 300 kHz were utilised. Image processing and analysis was conducted in Gwyddion by David Nečas and Petr Klapetek.^57^

#### SEM measurements

SEM micrographs were recorded on a LEO 1530 FE-SEM (Zeiss, Germany) with in-lens and SE2 detectors using an acceleration voltage of 3 kV. To enhance the conductivity on the wrinkle-templates they were covered with a 1.3 nm Pt layer using a HR208 sputter coater and a mtm20 thickness controller (Cressington Scientific Instruments).

#### Dip-coating

For dip-coating experiments a DC/D/LM system from KSV Instruments, Germany was used.

#### Plasma treatment

Oxygen plasma treatment was performed using a Flecto 10 from Plasma Technology, Germany.

#### Contact angle measurements

Contact angle measurements were performed using a DataPhysica OCA instrument from DataPhysics, Germany. The measurements were carried out on a hydrophilised PDMS substrate (0.2 mbar, 30 s) using a 0.5 mM CTAB solution. The contact angles of the applied droplets were measured after 150 s to ensure equilibrium. The measurements were repeated 4 times.

#### Image evaluation

Image evaluation was performed by a semi-automatic procedure in IGOR Pro by Wavemetrics, USA. First, the 8 Bit SEM images were corrected for maximum contrast and converted to binary data based on a grey value threshold that allowed for precise tracing of the boundaries of the particles. Particles touching the edges of the image were excluded from the evaluation. Next, the positions of each particle were located at the centres of the individual particle boundaries. The particle orientation was determined based on the weighted boundary-to-centre distances in the 8 Bit image. The relative angle deviations were calculated in reference to the mean angle of the complete image. The equation *S*_2D_ = 2 cos(*θ*)^2^ ¬ 1 was applied to calculate the two-dimensional order parameter *S*_2D_. The distribution of gap sizes was evaluated from the corrected 8 Bit images and the particle positions as an overlay. The individual particle lines were identified and represented by a linear regression of the centres of contributing particles. Gaps were evaluated manually from the intensity profiles along each particle line based on a threshold grey value (half maximum). The filling factor describes the one-dimensional allocation of space of each particle based on its projection onto the aforementioned linear regression of the respective particle line. The sum of all projections was set in relation to the total length of the regression line.

#### FDTD simulations

Simulations of the extinction cross-section spectra were done using commercial software from Lumerical Solutions, Inc. (FDTD Solutions, Version 8.7.3). We modelled the gold nanorods in water (index of 1.333). In agreement with the experimental peak position, we chose a cap radius of 10 nm and nanorod dimensions according to TEM analysis. The simulation for nanorods on substrates was performed for a single nanorod covered with a 2 nm layer BSA (index of 1.48)^14,58^ located on an ITO layer modified with a 2 nm roughness layer in air.^59^ For a broadband source simulation (total-field scattered-field source, 300–1300 nm), the FDTD software approximates the refractive index of the materials by a polynomial function. For the optical constants of Au, we applied a fitting of the experimental data by Johnson and Christy (JC) (6 coefficients, 1 imaginary weight: 0.211 RMS error).^60^ A simulation mesh size of 1 nm was chosen and the zero-conformal-variant mesh refinement was used. For the best simulation stability, the mesh area was chosen to be 50 nm larger than the existing structure in all three principle directions. All simulations reached the auto shut off level of 10^–5^ before reaching a simulation time of 150 fs. Anti-symmetric boundary conditions (BC) were used normal to the polarisation plane and symmetric BC are used parallel to the polarisation plane. In the radiation direction we used the perfect match layer (PML) in both directions BCs.
